# Chondrosarcoma Arising from the Posterior Iliac Crest Extending into the Spinal Canal

**DOI:** 10.1155/2021/5510075

**Published:** 2021-07-17

**Authors:** Kota Wada, Akio Sakamoto, Rei Kato, Takashi Noguchi, Takayoshi Shimizu, Bungo Otsuki, Koichi Murata, Shunsuke Fujibayashi, Shuichi Matsuda

**Affiliations:** Department of Orthopaedic Surgery, Graduate School of Medicine, Kyoto University, Shogoin, Kawahara-cho 54, Sakyo-ku, Kyoto 606-8507, Japan

## Abstract

Chondrosarcoma is a malignant tumor characterized by the production of a cartilage matrix. Extension into the spinal canal from the extracannular space is seen mainly for neurogenic tumors, but it is rare in nonneurogenic tumors. A 75-year-old woman suffered from sciatic pain and numbness in her lower left extremity. The diagnosis was of a low-grade conventional chondrosarcoma, which originated from the posterior ilium with an intraspinal extension at the level of the sacrum, compressing the cauda equina. The tumor extended further into the S1 sacral anterior foramen, in the shape of a dumbbell. The tumor was resected in several blocks posteriorly, and the dumbbell-shaped tumor in the S1 foramen was resected by widening the S1 foramen from behind. The posterior extension of the iliac tumor seemed prevented by the posterior sacroiliac ligament, and the tumor extended into the canal. Here, we report that the iliac chondrosarcoma extending into the spinal canal is rare for this tumor type. An understating of the tumor extension is important for planning the surgical strategy.

## 1. Introduction

Chondrosarcoma is a malignant tumor characterized by the production of a cartilage matrix [1]. Chondrosarcomas usually arise in the pelvis or long bones [2]. Conventional chondrosarcoma of the bone constitutes approximately 85% of all chondrosarcomas [2], and the pelvis is a commonly affected site [3].

Extension into the spinal canal from the extracannular space is seen mainly for neurogenic tumors, such as schwannoma or neurofibroma, but the finding is rare for nonneurogenic tumors. Nonneurogenic tumors reported to have an intracanal extension are hemangiomas, teratomas [4], and lipomas [5], all of which are benign. Spinal canal extension of chondroid tumors is rare. Chondroid tumors are usually periosteal enchondromas, which originate from a cervical vertebral body [6, 7]. Extension of a tumor from an iliac origin into the spinal canal would be rarer still, and to our knowledge, no such extension has yet been reported for chondroid tumors including chondrosarcomas.

Here, we report the case of a conventional chondrosarcoma originating from the ilium that extended into the sacral spinal canal. We also discuss the possible mechanism of the tumor extension pattern.

## 2. Case Report

**Figure 1:**
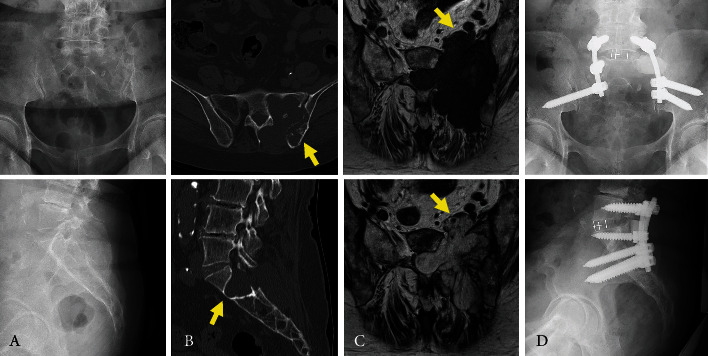
Chondrosarcoma in a 75-year-old woman. A plain radiograph showing a poorly specific finding in the sacrum (A). Computed tomography showing destruction of the bone in the left posterior ilium (arrow) ((B), top). The anterior cortex of the S1 vertebra was scalloped (arrow) ((B), bottom). Magnetic resonance imaging shows a lobulated lesion with homogenous low signal intensity on a T1-weighted image ((C), top) and high signal intensity on a T2-weighted image. The septal structure of each lobulated lesion had slightly low signal intensity on T2-weighted imaging ((C), bottom). The tumor is protuberant with a dumbbell shape in the enlarged S1 anterior foramen (arrows) (C). Plain postoperative X-ray radiograph, showing fixation of the L5-S-ilium (D).

A 75-year-old woman became aware of pain and numbness in her left extremity over 6 months before its initial assessment. The symptoms gradually worsened, causing difficulty in sleeping. A plain radiograph of the sacrum showed a slight abnormality of irregular cortex of the first sacral (S1) vertebra to the ala (Figure 1(A)). Computed tomography (CT) showed a tumor that occupied not only the left side of the S1 vertebra to the ala but also the spinal canal. Destruction of the bone was seen clearly in the left posterior ilium with a discontinuous cortex. The destruction was less obvious in the anterior cortex of the S1 vertebra, and the cortex was scalloped with remodeling, which is characteristic of benign bone lesions (Figure 1(B)). Magnetic resonance imaging (MRI) demonstrated a lobulated lesion in the left sacrum with a homogenous low signal intensity on T1-weighted images and a high signal intensity on T2-weighted images. The tumor in the spinal canal compressed the cauda equina. Furthermore, the tumor extended along the S1 nerve root to the anterior sacrum, with the shape of a dumbbell in the enlarged S1 anterior foramen (Figure 1(C)). A needle biopsy was obtained from the posterior ilium. The histopathological diagnosis was a low-grade conventional chondrosarcoma (grade I). Because bone destruction was seen mainly in the posterior ilium, the tumor was assumed to originate from the ilium extending to the sacrum. After careful observation of our patient, the posterior iliac chondrosarcoma was found to have occurred on the anterior surface of the iliac tuberosity.

The tumor was preferentially resected en bloc. However, the lesions were extensively located in the bone. Taking into consideration the age of the patient, the pelvic ring was preserved. The lesion was resected in several blocks, and the tumor remaining in the bone was curetted. The patient was placed prone during the surgery. The posterior component of the tumor at the extraspinal canal was resected with the affected ilium en bloc. A laminectomy extending from the lower part of the L5 (fifth lumbar) vertebra to the upper part of the S2 (second sacral) vertebra was conducted. The tumor in the left sacrum to the sacroiliac joint was removed by curettage while protecting the left S1 and S2 nerve roots. The left outer cortical bone of the S1 foramen was enlarged with a high-speed surgical burr, and the anterior part of the tumor was resected en bloc. After complete resection of the tumor, an L5 and S1 transforaminal lumbar interbody fusion (TLIF) was conducted. The defect at the left sacroiliac joint was filled with a low-porosity *β*-tricalcium phosphate (TCP) block, which has strong compression resistance (OSferion60; Olympus Terumo Biomaterials, Tokyo, Japan) (Figure 1(D)).

**Figure 2:**
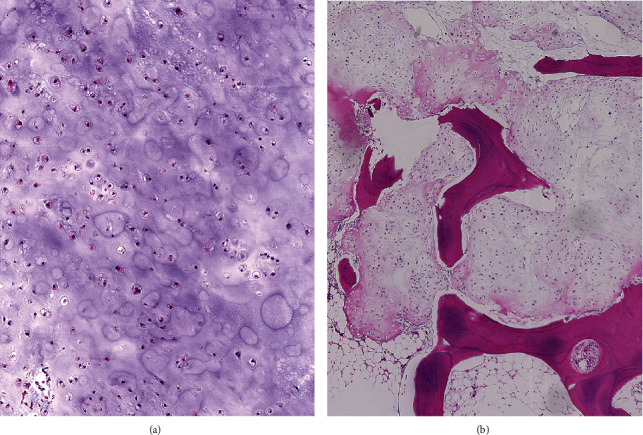
Grade I chondrosarcoma, moderately cellular and containing hyperchromatic, plump nuclei of uniform size (a). Infiltration through the marrow cavity with a “permeation pattern” is seen (b).

**Figure 3:**
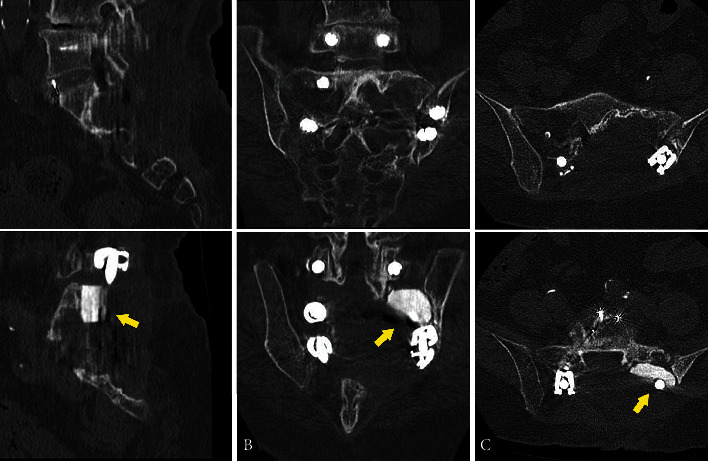
As of 10 months after the surgery, computed tomography shows no recurrence of the tumor and no dislocation of the implants including the *β*-tricalcium phosphate (yellow arrows): (A) sagittal; (B) coronal; (C) axial.

The final histopathological diagnosis of the resected material was conventional chondrosarcoma, grade I (Figure 2). At a 10-month follow-up, the patient still had numbness in her lower left extremity. However, previously strong sciatic pain had disappeared. Motor deficit complications did not occur. No recurrence of the tumor was seen 10 months after the surgery (Figure 3).

## 3. Discussion

MRI showed a lobulated lesion in the left sacrum with a low signal intensity on T1-weighted images and a high signal intensity on T2-weighted images, which was consistent with chondrosarcoma [8]. MRI successfully demonstrated the tumor extension into the spinal canal and S1 anterior foramen. CT as well as intraoperative findings suggested that the chondrosarcoma occurred on the anterior surface of the iliac tuberosity. The posterior surface of the iliac tuberosity to the sacrum was covered by the sacroiliac ligament. The tumor from the posterior ilium grew under the ligament and extended into the spinal canal. Therefore, we considered that the posterior sacroiliac ligament and other surrounding ligaments prevented the posterior extension and allowed the tumor to progress to the spinal canal and anterior part from the sacral foramen.

Pelvic bone tumors, including chondrosarcoma, are common in the ilium [9], and resection of the ilium bone tumors, with or without partial sacral resection, is conducted [10]. A complete resection of the tumor extending to the iliosacral joint may lead to disruption of the pelvic ring [11]. If this occurs, reconstruction after the resection is necessary to stabilize the pelvic ring and preserve limb function [12]. In the present case, the iliosacral joint was preserved partially, and the pelvic ring was preserved completely.

Chondrosarcomas are graded histologically from I to III. Grade I chondrosarcomas often closely resemble benign enchondroma [8]. The characteristic feature distinguishing chondrosarcomas from enchondromas is the demonstration of tumor infiltrates through the marrow cavity in a “permeation pattern” [13]. The histological grading is associated with the rate of local recurrence and of survival of the patients [13, 14]. Local recurrence of pelvic chondrosarcomas is reported to be 38%, of which 30% are grade I, 33% are grade II, and 56% are grade III. The risk of disease-related death was 3% for grade I, 33% for grade II, and 54% for grade III [15]. It has been assumed that low-grade chondrosarcoma has the potential to reoccur but is less affected by the overall survival, at least at midterm follow-up [16].

The incidence of local recurrence is significantly affected by the adequacy of the surgical margin in pelvic chondrosarcoma [11], and a wide surgical margin is recommended regardless of the grade when resecting a pelvic chondrosarcoma [15]. A margin of 1 mm or more appears to be sufficient in the pelvic chondrosarcoma in an attempt to achieve a negative margin [3]. In the presently reported case of grade I pelvic chondrosarcoma, intralesional resection was conducted preserving the iliosacral joint. Intralesional margins seem to be acceptable for grade I chondrosarcomas [14]. However, careful follow-up will be necessary in the present case to check for recurrence because of the intralesional resection used.

In the present case, the lesion extended to the anterior S1 foramen with a dumbbell shape associated with enlargement of the foramen. The term “dumbbell tumor” is descriptive only; its pathological nature was not considered. However, “dumbbell tumors” are assumed to cause pressure erosion of the bone, without infiltrating it. During the resection, the S1 anterior foramen was widened to the tumor size from behind, and the entire lesion was resected in one piece. An additional anterior approach was an option for its complete resection. However, the iliac vein was located adjacent to the tumor. Compared with the posterior approach taken, an anterior approach would be more invasive with longer operative time and more bleeding. In addition, the surface of the sacrum was covered by the sacroiliac ligament, and the protuberant tumor was considered to be located beneath the ligament. Therefore, to obtain an operative field sufficient for tumor resection was considered to be difficult.

## 4. Conclusion

The tumor extension pattern was considered when the resection was planned, especially for the spinal canal component and the dumbbell type tumor through the S1 nerve foramen. An understanding of the tumor extension is important for planning the surgical strategy.
